# A Critical Review on Selected External Physical Cues and Modulation of Cell Behavior: Magnetic Nanoparticles, Non-thermal Plasma and Lasers

**DOI:** 10.3390/jfb10010002

**Published:** 2018-12-24

**Authors:** Barbora Smolková, Mariia Uzhytchak, Anna Lynnyk, Šárka Kubinová, Alexandr Dejneka, Oleg Lunov

**Affiliations:** 1 Institute of Physics of the Czech Academy of Sciences, 18221 Prague, Czech Republic; smolkova@fzu.cz (B.S.); uzhytchak@fzu.cz (M.U.); lynnyk@fzu.cz (A.L.); dejneka@fzu.cz (A.D.); 2 Institute of Experimental Medicine of the Czech Academy of Sciences, 14220 Prague, Czech Republic; sarka.k@biomed.cas.cz

**Keywords:** magnetic nanoparticles, non-thermal plasma, lasers, cytotoxicity, cell signaling

## Abstract

Physics-based biomedical approaches have proved their importance for the advancement of medical sciences and especially in medical diagnostics and treatments. Thus, the expectations regarding development of novel promising physics-based technologies and tools are very high. This review describes the latest research advances in biomedical applications of external physical cues. We overview three distinct topics: using high-gradient magnetic fields in nanoparticle-mediated cell responses; non-thermal plasma as a novel bactericidal agent; highlights in understanding of cellular mechanisms of laser irradiation. Furthermore, we summarize the progress, challenges and opportunities in those directions. We also discuss some of the fundamental physical principles involved in the application of each cue. Considerable technological success has been achieved in those fields. However, for the successful clinical translation we have to understand the limitations of technologies. Importantly, we identify the misconceptions pervasive in the discussed fields.

## 1. Introduction

The employment of physics principles to biomedical field of research has made an outstanding contribution in various applications related to diagnosis and treatment of different pathological conditions [1,2]. Indeed, a strong relationship always existed between physics and medicine. For example, the first formal report by Röntgen of his discovery of X-rays was made to a medical society. Despite the sophistication and specialization demanded by modern physics and modern biomedicine, the fields maintain a strong interaction. Indeed, modern biomedicine owes much to modern physics. It is hard to imagine modern hospital without a magnetic resonance imaging (MRI) scanner, diagnostic radiology, angiography, computed tomography, and ultrasound. Further amalgamation of research, application, cooperation and understanding of physics and biomedicine is expected to facilitate development of novel diagnostic and therapeutic strategies.

Researchers continue to develop safe and effective implementation of physics-based technology that bring great impact in the advancements of biomedical sciences [1,2]. We will not discuss here all modern physics-based biomedical approaches. We will focus instead only on selected novel, provocative, leading edge developments. In this review article, we overview actively researched topics of application of external physical cues intended for novel diagnostic and therapeutic strategies which encompass:

- Using high-gradient magnetic fields in nanoparticle-mediated cell responses;
- Non-thermal plasma as a tool for non-specific bacterial killing;
- Highlights in understanding of cellular mechanisms of laser irradiation.

We selected those topics of research, because they are relatively new, actively developed by scientific community and hold great promises in constructing a basement for future therapeutic modalities. However, those technologies need to prove if they can actually meet the high expectations in evidence based medicine (EBM) controlled health care systems [3,4]. Such bleeding edge research, as it often happens with frontiers in sciences, is hampered by shortcomings in experimental design and sometimes by a lack of reproducibility [5,6,7,8]. This review aims to identify gaps in our understanding of underlying biochemical mechanisms in selected physics-based biomedical approaches. In the absence of a hypothetical mechanism to guide experimental design, proper adjustment and control of the experimental parameters are usually precluded. Only the knowledge of the spatiotemporal mechanisms of the induced effects will enable the deliberate exploitation of such signals, e.g. for the remote control of cellular signaling processes. Thus, in this review we discuss current challenges and perspectives in each section.

## 2. Using High-Gradient Magnetic Fields in Nanoparticle-Mediated Cell Responses

Nanoparticles (NPs) found their applicability in variety of biomedical applications such as, probes for cell and subcellular structure labeling, as well as for drug and gene delivery [9]. Magnetic nanoparticles (MNPs) are extensively studied in a variety of biological and medical applications, such as magnetic resonance imaging (MRI), drug delivery and studies of cell mechanics [10,11]. It turned out that rapid and effective loading of cells with MNPs is crucial for a wide variety of biomedical applications, such as cell labeling for in vivo imaging, guided cell delivery, gene/drug delivery [12,13,14,15]. Hence, approaches to increase the efficiency of magnetic cell labeling are still required. In order to improve the therapeutic impact and to reduce off-target effects, it is feasible to take an advantage of the combined use of MNPs and external magnetic fields.

Static magnetic fields generated by permanent magnets were found to be an easy option to affect MNPs. We will not discuss in great details here biological effects of magnetic fields. This research field is highly disputable and has been extensively reviewed and discussed previously [6,16,17]. Many reported effects of magnetic fields on tissues have not been replicated [18,19,20,21]. In other cases, attempts to replicate published effects have not been successful [18,19,20,21,22,23,24]. Specifically, attempts to directly reproduce biological effects of magnetic fields were unable to repeat the initial findings, e.g.: induction of chromosome defects, increase of *MYC* and β-actin transcription, activation of cellular motility, Ca^2+^ oscillations [18,19,20,21,22,23,24]. The detailed list of the effects of magnetic fields on tissues, that have not been replicated or did not demonstrate consistent results, can be found in previously published comprehensive reviews [6,20,22,23]. Moreover, randomized double-blind trials and meta-analysis of randomized trials showed no support in the use of static magnets for pain relief, and therefore magnets cannot be recommended as an effective treatment [21,25]. Of note, only an exceptionally high gradient of magnetic field (>10^5^ T/m) might exert some potential biological activity [16,17]. In fact, biological matter is affected weekly by magnetic fields. Indeed, due to the fact that magnetic fields can penetrate through tissues essentially undisturbed, they are used in clinical practice for whole-body medical imaging [26]. Definitely, there is a need for additional rigorous studies that will thoroughly verify and firmly establish biological effects of magnetic fields. However, combination of magnetic field with magnetic nanoparticles has been shown to be potentially useful in biomedical applications [12,13,14,15].

Numerous studies have reproducibly shown that MNPs are particularly useful for the manipulation and control of specific cellular functions under application of external magnetic fields. First of all, utilization of functionalized MNPs coupled with nucleic acids and guided by an external magnetic field to the targeted cells facilitates the introduction of nucleic acids into the cells [27]. This procedure is called magnetofection. Indeed, various nucleic acids formulations have been successfully employed with MNPs, e.g., pCIKlux plasmid DNA, pCI plasmid DNA, siRNA (anti-GFP or anti-survivin), and GFP plasmid (for review see [27]). Nucleic acid vectors are associated with magnetic nanoparticles then permanent magnets are placed below cell culture dishes in order to attract the vector to the cell surface [15,27]. High-gradient magnetic fields that are applied in magnetofection in the vicinity of the cells typically reach 70–250 mT and a field gradient of 50–130 T/m [15,27]. Magnetofection is attractive due to its relative simplicity. It requires suitable magnetic nano- or micro-particles and appropriate magnetic devices. We summarized MNP and magnetic devices which showed high efficacy in gene delivery by magnetofection (Table 1). Applicability of magnetofection was very straightforward that it resulted in commercialization of magnetofection tools together with standardized application protocols for various vector types and cell culture formats (OZ Biosciences, Marseille, France, http://www.ozbiosciences.com; Chemicell, Berlin, Germany, http://www.chemicell.com). MNP-based transfection could be applied virtually any cell type due to its endocytic uptake. This approach is very useful for cell lines that are difficult to transfect by conventional methods [28,29].

It is worth noting here, that nanoparticles depending on the particle size and surface treatment may enter cells via different pathways [38,39]. It has been found that NPs uptake by living cells can be classified into following major pathways: phagocytosis, micropinocytosis, clathrin-mediated endocytosis, caveolin-dependent endocytosis, and non-specific interactions [38,39,40]. Phagocytosis is primarily used to uptake dead cells, cell debris, and pathogens. Macropinocytosis is an actin-regulated process that involves engulfment of a large quantity of extracellular fluid and particles through plasma membrane ruffling [38,39,40]. In clathrin-mediated endocytosis, receptor-ligand binding triggers the recruitment and formation of “coated pits” (clathrin) on the cytosolic side of the plasma membrane. Caveolin-dependent endocytosis requires the assembly of the hairpin-like caveolin coats on the cytosolic side of the plasma membrane, forming a flask-shaped caveolae of ∼50–80 nm in diameter [38,39,40].

Application of an external magnetic field gradient to MNPs causes magnetization and subsequent movement due to the force acting on it [41]. An applied force exerted on the particles results in NPs movement toward the highest field strength. Being injected in the blood stream, MNPs have to overcome the competing forces exerted on the particles by the blood compartment [41,42]. Theoretical studies of the hydrodynamic conditions of MNP targeting in combination with experimental work show, that for most magnetic carriers, the field strength (flux density) at the target site should be of the order of 200–700 mT with gradients along the z-axis of approximately 8–100 T/m [41,42,43,44]. Computational modeling reveals that when the magnetic forces exceed the linear blood flow rates in arteries (10 cm/s) or capillaries (0.05 cm/s), the MNPs will be retained at the target site and may be internalized by the cells [41,42,43,45]. However, one should bear in mind that modeling is somewhat idealized and limited [41,42]. Thus, results of computational modeling have to be interpreted with caution [41,42]. It is worth noting here that a pulsed magnetic field can be used to effectively enhance the cellular uptake and transport of MNPs across cell barriers relative to a constant magnetic field [46]. This effect is achieved by promoting MNPs accumulation while minimizing magnetically induced aggregation at the cell surface [46].

A number of studies report that magnetic field could be effectively utilized for MNPs-mediated targeted gene and drug delivery in cancer therapy [47,48], including delivery of therapeutic plasmid for treatment of melanoma [49] and adenocarcinoma [50]. Indeed, drug delivery and cell therapy utilizing magnetically guided MNPs were successfully tested on models of retina [51] and corneal endothelium [52,53].

One should bear in mind that in order to obtain sufficient magnetofection efficacy with high cell survival rate, the properties of nanoparticles, magnetic field strength and gradient are crucial. The magnetic field gradient exerts force on MNP that could be derived as *F* = *p_m_*·(*dB*/*dz*). Here, *p_m_* is the magnetic moment of the MNP. If MNP is saturated by magnetic field, its magnetic moment is *p_m_* = *M_s_∙V*, where *V* is the MNP volume and *M_s_* is the saturation magnetization of nanoparticle. Rough estimations of the magnetic force acting on the most widely used magnetite-based (*M_s_* = 412 kA/m [54]) iron oxide nanoparticle of average diameter ~50 nm give the force value of about 0.003 pN. Of note, MNP aggregation under external magnetic field plays a critical role in determining the behavior of the nanoparticles [55]. However, mechanical forces occurring in nature (such as channel gating force, traction forces/pulling forces by actin fibers, tissue deformation forces) are in the range of 0.2–100 pN [56]. Specifically, mechanical pulling force produced by endocytosis is in the range of 0.1–3 pN [57]. Moreover, to activate intracellular signaling and trigger endocytosis one needs to reach forces of about 1–5 pN [58]. How is it possible that magnetic fields exerting force of about 0.003 pN on a single nanoparticle might have any biological effect? Indeed, upon contact with biological fluids, nanoparticles interface with various biomacromolecules [59,60,61]. Therefore, despite the fact that magnetic particles used in magnetofection and other applications are nanosized, the magnetic complexes form aggregates in cell culture media with sizes of several hundred nanometers to microns [60,62]. For such big clusters magnetic force reaches already 1–50 pN [56,63,64].

**Figure 1:**
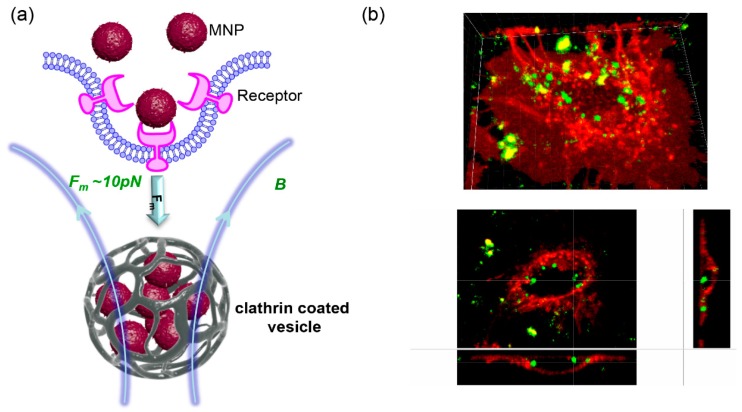
(**a**) Scheme of magnetically-assisted magnetic nanoparticles’ (MNPs’) uptake through clathrin mediated endocytosis; and (**b**) 3D reconstruction MNPs’ uptake in the presence of magnetic pulses together with confocal orthogonal images. Cell membranes were labeled with CellMask™ Deep Red (red); nanoparticles are green.

However, for effective magnetic cell labeling, magnetofection, or triggering cellular processes, several hours of magnetic fields exposure might be needed [15,55,56,65]. Application of pulsed magnetic fields may enhance nanoparticle uptake and result in a reduction of incubation time [14]. Another complementary strategy potentially applicable to increase the magnetic response is to improve our ability to generate strong magnetic field [66]. Indeed, upon application of a high intensity (7 T) short pulse width (~15 µs) magnetic field one can dramatically enhance endocytosis of MNPs (Figure 1) [66].

We summarized recent studies that have demonstrated how MNPs can serve to convert the external signal of magnetic fields into biological events in Table 2.

As one can clearly see from the Table 2, in order to trigger any biological events magnetic fields must exert forces on nanoparticles laying in the range of naturally occurring mechanical forces of about 0.2–100 pN. As a matter of fact, all studies, discussed here, used either superparamagnetic or ferromagnetic nano(micro)particles. It is logical to have highly magneto-responsive material in order to manipulate it using magnetic fields. It is worth noting here, that a careful and quantitative examination of the physical mechanisms of magnetic fields action on MNPs has to be seriously considered [26,55,56,75,76,77]. In particular, some of the recent studies proposed peculiar activation mechanisms of biological reactions utilizing endogenous paramagnetic ferritin nanoparticles that have been affected by external magnetic fields [78,79]. Those studies have been heavily questioned [26]. Indeed, application of basic laws of physics to the experimental conditions reported revealed that the force and/or torque exerted by the endogenous ferritin NP on the attached receptor/channel are 4 to 9 orders of magnitude lower than those due to thermal fluctuation noise [26]. It was shown that the paramagnetic nature of protein complexes precludes execution of biological response [26].

To conclude this part, utilizing high-gradient magnetic field in combination with MNPs without any doubt bears true potential and will be an active field of research in the forthcoming years. However, we need to overcome numerous challenges and open questions. First of all, we should look deeper into the mechanisms of biological processes triggered by application of magnetic field on MNPs. Secondly, we have to rationally define inputs and measurable outputs to dissect the molecular circuits underlying the cellular response to external cues.

## 3. Non-Thermal Plasma as a Tool for Non-Specific Bacterial Killing

Over the last years, antibiotic resistance and rapid growth of nosocomial infections are one of the biggest problems to global health and food safety [80]. The evolution of drug-resistant bacteria has prompted the interest of scientist in the development of new effective antibiotics [81,82] or other alternatives to conventional treatment, such as phage therapy [81], honey-based templates/biomaterials [83,84], ion-based strategies [85,86,87]. In last decades, physical approaches UV irradiation [88], ultrasounds [89], high pressure processing [90] and the non-thermal plasma (NTP) [91,92,93] emerged as potential alternative to antibiotics. It this part of review we will focus on non-thermal plasma (synonyms: cold plasma, low temperature plasma, atmospheric pressure low temperature plasma).

Non-thermal plasma with its anti-microbial effects showed to be very effective tool in bacterial decontamination [93,94,95,96]. NTP is a partially ionized gas, containing chemically active complex that includes reactive oxygen and nitrogen species, electrons, UV photons, ions, electrons, and free radicals [97]. Generally, plasma represents a neutral charged mixture of atoms and charged particles. According to temperature of the electrons plasma can be divided into thermal (T_e_ 10^6^–10^8^ K) and non-thermal (T_e_ 300–10^3^ K) [97]. Only thermal plasma, where the temperature of electrons (T_e_) and heavy particles (T_n_) is approximately the same, will reach the thermodynamic equilibrium [97]. Contrary, in non-thermal plasma the heavy particles’ temperature is much lower in comparison with T_e_. Thus, the temperature of whole complex is low, under 40 °C and such plasma can be use in biomedicine [97].

**Figure 2:**
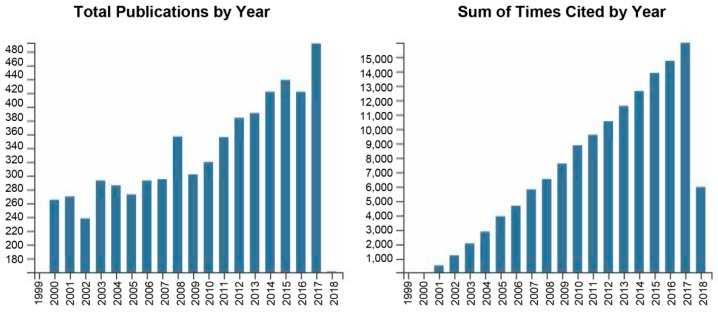
Graphs of Total Publications and Sum of Times Cited by Year for the period of 2000–2018, taken from Web of Science database (search has been done by following settings: TOPIC: (non-thermal plasma) OR TOPIC: (cold plasma) OR TOPIC: (cold atmospheric plasma) OR TOPIC: (low temperature plasma); Refined by biomedical sciences).

Although, the bactericide effects of ionized gas have been known for many years, first results showing the use of cold plasma for bacterial sterilization were published by Laroussi in 1996 [98]. From that time, the number of publication is rapidly and constantly growing (Figure 2). Indeed, plasma technology found the utilization in many biomedical applications from sterilization of medical equipment [99], wound healing [100,101] dental hygiene [99,102] blood coagulation [103] and cancer treatment [104] to food industry [96].

NTPs can be produced by discharges in virtually any desired gas or mixture of gases in order to produce a ‘chemical cocktail’ of atoms, ions and molecules for biomedical applications [97]. The most common technological developments with relevance to health care are dielectric barrier discharge (DBD) and atmospheric pressure plasma jet (APPJ) [97]. Both approaches have advantages and disadvantages. In DBD carrier gas moves between two flat metal electrodes covered with dielectric material, where is ionized to create plasma. The high-voltage electrode is needed for plasma generation, whereas the second one is grounded [97]. DBD uses the skin (or other tissue) as an electrode so that the current produced has to pass through the body [105]. Interestingly, in 2006, Fridman et al. replaced one of the electrodes by using an object with high charge storage capacity, namely floating electrode (FE) [106]. In this case, plasma is generated in between insulated electrode and living tissue, which has the required capacity for charge storage and can be used as so-called floating electrode [106]. The main advantage of FE-DBD is direct application on living tissue without thermal or chemical damages [106,107]. Indeed, DBD has been shown as very promising tool for blood coagulation and tissue sterilization [103]. However, high voltage applied directly to the cells or tissue may affect the cellular functions by direct stimulation with electric current. In contrast, by the indirect method, plasma is produced between two electrodes and are then transported to the area of application entrained in a gas flow [105]. In the past few years, different devices have been developed, ranging from very narrow plasma needles to larger plasma torches, for review see [97].

A number of studies showed consistently the antimicrobial effects of non-thermal plasma against different types of microorganisms [97]. An assortment of recent inactivation results achieved with different types of NTP is summarized in Table 3.

**Figure 3:**
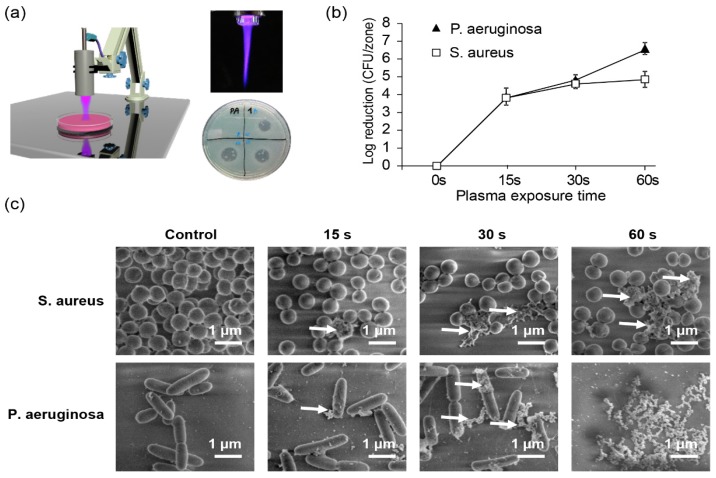
Inactivation of bacteria by non-thermal plasma. (**a**) Scheme of non-thermal plasma (NTP) treatment, image of plasma torch and example of antibacterial NTP effects in vitro; (**b**) Time-dependent inhibition of two bacterial strains *S. aureus* and *P. aeruginosa*; (**c**) Scanning electron micrographs of untreated and helium NTP- treated bacteria *S. aureus* and *P. aeruginosa* [100] (CC BY license 4.0, 2017, Scientific Reports).

Nowadays, it is widely discussed in literature that the mechanism of NTP action is based on the synergism of biologically active particles, mostly reactive oxygen (ROS), nitrogen species (RNS) and ions [93,94,95,96]. In fact, the bactericidal effect of NTP greatly depends on various physical parameters, including input voltage and current, type of gas, flow rate, treatment time, and method of NTP generation. The environmental factors and microbial properties also play an important role in resulted NTP bactericidal efficacy [93,94,95,96]. It is worth nothing that several lines of research revealed presence of a variety of bactericidal agents in plasma [93,94,95,96]. However, the detailed mechanism of bacteria inactivation triggered by NTP still remains unclear. Studies depicted following potential bactericidal plasma-generated agents: UV light, ROS, RNS, and ion flux [93,94,95,96]. An example of NTP torch and bacteria eradication upon NTP treatment is shown on Figure 3.

It is well-known that UV light at 260 nm may induce modification in DNA, especially formation of pyrimidine dimers and pyrimidine-pyrimidone(6-4) photoproducts [123]. Over the years, the bactericide effects of UV were used in sterilization. Therefore, it is logical to assume, that the destruction of DNA triggered by plasma generated UV photons could be a potential mechanism of antimicrobial inactivation [124]. However, we and others showed very low or negligible effect of UV light generated by plasma on bacterial deactivation [114,125,126]. In our previous study, we used sapphire glass by NTP treatment of 4 different bacterial strains to exclude the UV influence. We didn´t observe any effect of UV on *E. coli*, *S. aureus*, or *B. subtilis* and only weak on *P. aeruginosa* post plasma treatment using the glass [114]. Contrarily, others depicted UV light produced by plasma as major agent of bacterial inactivation [127,128]. However, we used He plasma [114], whereas Ar was used as carrier gas for plasma generation in [127,128]. Indeed, it is becoming evident that NTP bactericidal effects dramatically depend on chemical composition of plasma [93,94,95,96]. In fact, chemical composition of plasma and plasma-treated liquids may grossly vary, depending on the carrier gas that forms the NTP [129,130,131,132]. This issue sheds light on first challenge in the field. It is hard to compare different studies, because they use different gases to produce NTP. It is relatively easy to manipulate plasma chemical composition using distinct gases, such as helium (He), argon (Ar), nitrogen (N_2_), ambient air, or a mixture of gases [97]. Consequently, the biological responses to NTPs vary dramatically depending on the physical and chemical characteristics of plasma [129,130,131,132]. Second major challenge in the field is that the voltage producing NTP discharges varies greatly (0.5–100 kV) depending on the type of plasma device [97]. Additionally, voltage frequencies of NTP generators vary enormously [97]. This, in turn, brings another degree of variability in triggering biological responses by NTP. Hence, it is of great importance to pay careful attention when comparing bactericidal effects triggered by plasma originating from discharges produced by different voltages.

Indeed, power measurements of UV production by plasma system (of discharges lower than 10 kV) showed that the power density of the emitted UV radiation for air-based NTP is lower than 1 μW/cm^2^ and for He NTP—3 ± 1 μW/cm^2^ [129,130,131,132]. These values are at least one order of magnitude lower than the minimal power density needed to have any effect on living cells [133]. Moreover, we and others have deliberately shown that the UV radiation is not the dominant biological agent of non-thermal plasmas of such ion density and energy [114,125,126].

Most studies agree that NTP generated by a voltage of >10 kV completely destroys the bacteria [93,94,95,96]. The electrostatic disruptions followed by plasma treatment are the most studied [134]. Accumulation of plasma generated charged particles like ions and electrons may have the crucial role in the rupture of bacterial membrane [93,94,95,96,134]. The bombardment of charged particles on the outer surface of bacterial membrane could overcome the tensile strength of the membrane and lead to bacterial death [116,134]. Specifically, under NTP treatment mechanically rigid bacterial wall structures crack as a result of internal electrostatic pressure raise due to ions accumulation [116,134]. This is one of potentially feasible antibacterial mechanisms of NTP action against bacteria. Such bacterial wall cracking is accompanied with damage of the bacterial cell wall and the outer membrane, leading to leakage of cellular components, such as nucleic acid and ATP [135,136]. It is worth noting that, especially by direct plasma treatment charged particles play a significant role is sterilization [137,138]. Fridman et al. observed better antimicrobial efficacy by using direct mode [137], probably due to the generation of strong electric field strength on bacteria. However, the physical mechanisms along cannot explain the inactivation of bacteria by NTP.

Another biologically active components of NTP are ROS. A burst of studies shows that ROS might be one of the major factor in microbial pathogens inactivation by NTP treatment [93,94,95,96,97,108]. Moreover, ROS and their chemical solutions, especially H_2_O_2_, are widely used for wound disinfection and sterilization in clinic [139]. Generally, ROS are responsible for essential cellular processes and act as signaling molecules in different organisms, from bacteria to human [140]. Indeed, the cellular responses to ROS depend on their intracellular concentration.

Oxidative damage of membranes, following ROS accumulation and destruction of intracellular compartments is one of the possible explanation for bacterial inactivation by NTP [109,117]. Due to close location to cell surface, membrane lipids are the most susceptible to plasma-generated ROS [141]. Interaction of plasma-generated ROS with peptidoglycan and polysaccharide may lead to damage of membrane structures [142]. Joshi et al. showed in their study morphological changes of the *E.coli* membrane as well as lipid peroxidation and DNA damage after plasma exposure [109].

Number of studies proposed different inactivation mechanism and higher resistance of Gram-positive bacteria to plasma in comparison to Gram-negative ones [114,116,121,122]. As an example, one can clearly see higher efficacy in deactivation of *P. aeruginosa* than in *S. aureus* after 60 s with He plasma treatment (Figure 3b). Only after 30 s of plasma treatment one may notice first disruptions in both studied strains. 60 s of NTP exposure leads to completely disrupted *P. aeruginosa*, whereas certain fraction of *S. aureus* is still intact (Figure 3c).

The cell walls of Gram-negative bacteria are covered by tiny layer of peptidoglycan and lipopolysaccharide membrane while Gram-positive bacteria consist of peptidoglycan with thick and strength structure [143,144,145]. Han et al., based on their results, suggested possible mechanism of NTP- action on bacteria [117]. In case of Gram-negative bacteria, the major target of plasma-generated ROS is cell envelope. Therefore, cell death occurs in response to peroxidation of membrane lipids, following cell leakage. Contrary, in Gram-positive bacteria higher penetration and accumulation of ROS lead to destruction of intracellular compartments and DNA without cell leakage. However, in another study the gram-positive *L. monocytogenes* strains were more vulnerable than gram-negative *E. coli* [122]. Thus, the NTP effect is highly dependent on studied microorganism and further investigations are necessary to clarify these controversies.

It is widely accepted that generation and accumulation of specific ROS can induce programmed cell death (PCD) in mammalian cells [146]. In recent years, increasing number of evidences indicates that programmed cell death also occurs in bacteria [147,148]. Could plasma-generated ROS trigger a programmed cell death in bacteria? Depending on the plasma dose and voltage value producing the plasma discharges, NTP may trigger either programmed cell death or physical destruction of the bacteria [93,94,95,96]. We observed the specific apoptotic physiological hallmarks, such as phosphatidylserine exposure on the cell membrane and increased expression of proteins with caspase-like substrate specificity [116]. Moreover, another study has shown the NTP- induced PCD in microorganisms [149]. Li et al. explained the *Microcystis aeruginosa* inactivation by intracellular accumulation of plasma- produced ROS leading to apoptosis [149]. However, more intensive studies are required to clearly explain the impact of NTP on programmed cell death in bacteria.

In conclusion, non-thermal plasma seems to be very effective and promising tool in inactivation of wide range of bacteria belonging to various morphological groups (Table 3). However, still there are challenging questions needed to be explained. The most serious problem represent that the exact mechanism is still not completely understood. Moreover, the effects of direct plasma treatment on the living cells range from the growth promoting response to apoptosis or even necrosis [129,150,151,152,153,154]. Thus, the dose, technical and physical parameters are crucial in plasma treatment. Pilot clinical studies of NTP devices showed promising results in skin and wound decontamination of multidrug-resistant bacteria [155,156]. However, experimental medicine must continue to dig into target identification and mechanism of action of therapies. Bactericidal plasma applications require well defined and controlled interactions between non-thermal plasma and living cells. Furthermore, the poor penetration depth and short working distance limit the other utilization of NTP for clinical transition. Nowadays, big attention is paid on application of plasma-activated liquids (PAL), either water, PBS, medium or other solutions. In recent years, number of studies are showing positive effects of PAL in different plasma field, from bacterial inactivation to cancer treatment [119,157,158]. Further studies are necessary to analyze the effects of such solutions very carefully. In the near future, the interdisciplinary cooperation of physicist, chemists, biologists, engineers, and doctors is necessary for development of international standards for plasma field. Thus, such a standardization of exposure mode might be useful by implementation of effective and safe plasma devices into in-field biomedical applications.

## 4. Highlights in Understanding of Cellular Mechanisms of Laser Irradiation

Light plays a crucial role in important biological processes directly related to a human health, such as: vision, vitamin-D metabolism, circadian rhythm, and psychosocial state [159]. Thus, it is not quite a surprise, that light has been utilized in different clinical applications: phototherapy, photodynamic therapy (PDT) or skin rejuvenation [160,161,162,163]. Research on potential biomedical utilization of low power red and near-infrared (NIR) lasers are gaining steadily increasing attention [162]. In particular, low power red and near-infrared (NIR) lasers showed some beneficial effects in a wide range of treatments from ophthalmology to oncology [161,162,164,165]. Low-power laser irradiation of red light (600–680 nm) has been shown to modulate various biological processes, such as cell proliferation and differentiation [166], cell viability and motility [167], and cell apoptosis [168,169,170]. Clinical application of low power red and near-infrared light has become a very active research area [159]. Indeed, low power red and near-infrared light treatment showed a potential to promote wound healing, hair growth, tissue regeneration or reduce pain and inflammation [162,165,171,172,173]. Such therapeutic modality was termed as Low Level Light/Laser Therapy (LLLT) [162,165,171,172,173]. Although LLLT became widely used to treat a variety of ailments, it remains controversial as a therapy [159,174,175,176]. It is worth noting here, that the applicability and current state of LLLT were reviewed previously [162,177].

Indeed, photons interact with biological tissue via various processes that can be broadly generalized into scattering and absorption. The propagation path, polarization and spectrum of incident light can be dramatically affected by scattering. The states of the scattered light can be assessed and mapped for diagnosis and imaging. The energy of photons is converted to electronic or vibrational energy during the light absorption. Some of that energy can be re-emitted through luminescence (for example, fluorescence), inelastic scattering, or acoustomechanical waves [159]. On the other hand, the tissues and cells can be affected by photoexcitation of intrinsic molecules or exogenous light-sensitive agents in different ways, via the generation of heat (photothermal), chemical reactions (photochemical) and biological processes (photobiological or optogenetic) [159]. In this part of our review, we critically overview current knowledge of cellular mechanisms of photobiomodulation during low-power laser irradiation. Photobiomodulation referred here as biological effects triggered by laser light that does not result in heating.

Precise biochemical mechanisms underlying the photobiomodulation exerted by red or NIR light are not yet well established [162,177,178]. From literature analysis, it appears that red light photobiomodulation has a wide range of effects at the molecular, cellular, and tissue levels [159,162,177,178]. There are some evidences that light might affect the mitochondria [168,170] resulting in increase of ATP production [179]. Additionally, photobiomodulation results in ROS accumulation and the induction of transcription factors [180]. There is a number of transcription factors that are regulated by changes in cellular redox state: redox factor-1 (Ref-1) dependent activator protein-1 (AP-1), nuclear factor kappa B (NF-κB), p53, activating transcription factor/cAMP-response element–binding protein (ATF/CREB), hypoxia-inducible factor (HIF)-1, and HIF-like factor [180].

Interestingly, there is some evidence that immune cells might be affected by photobiomodulation [181]. Specific wavelengths of light were shown to trigger mast cell degranulation [181], which is known to induce the release of the pro-inflammatory cytokine TNF-α from the cells [182]. This may result into increased infiltration of the tissues by leukocytes. In some studies, photobiomodulation resulted in proliferation, maturation, and motility enhancement of fibroblasts, and increased the production of basic fibroblast growth factor [183,184].

How can photobiomodulation possibly work? There are some studies that point to a chromophore within mitochondria being the initial target of photobiomodulation. Radiation of tissue with light might result in mitochondrial products increase such as ATP, NADH, proteins, and RNA [162,185]. It is plausible to propose some relevant chromophore by matching the action spectra for the biological response to light in the NIR range to the absorption spectra of the four membrane-bound complexes identified in mitochondria [162]. It was proposed that cytochrome c oxidase is the crucial chromophore in the cellular response to photobiomodulation [159,162,185]. Cytochrome c oxidase is a large transmembrane protein complex, consisting of two copper centers and two heme–iron centers, which is a component of the respiratory electron transport chain [186]. The electron transport chain transfers high-energy electrons from electron carriers through a series of transmembrane complexes to the final electron acceptor, generating a proton gradient that is used to produce ATP. Therefore, identification of cytochrome c oxidase as crucial chromophore in photobiomodulation is very attractive and can be used for explanation of alleged biological effects of light [159,162,185,187,188].

It is worth noting here that properties of low level lasers used for photobiomodulation are:

(a) Power output of lasers being 0.001–0.1 Watts.
(b) Wave length in the range of 300–10,600 nm.
(c) The optical intensity (defined as the optical power per unit area, commonly measured in W/cm^2^) of 0.01–10000 W/cm^2^ [159,189].

One may sub-divide studies into two groups. First group investigates low power (0.001–0.1 W) and low optical intensity (<10 W/cm^2^) laser photobiomodulation [159,162,177,178]. Second does research on low power (0.001–0.1 W) but high optical intensity (>>10 W/cm^2^, ~kW/cm^2^) lasers [159,168,170,190,191,192]. Interestingly, research on low power and low optical intensity laser photobiomodulation shows bewildering biological effects ranging from cancer killing to cell proliferation and differentiation [166], cell viability and motility [167], cell apoptosis [162,177]. We cannot review here all studies in this direction. They are too many. However, several reviews summarizing low power and low optical intensity laser photobiomodulation effects exist [162,177]. There is one major thing that unites those studies. Such studies show “positive” biological effects in vitro. However, alleged biological reactions are too broad, ranging from cell death to proliferation and maturation of progenitor cells [162,177]. Indeed, when it comes to clinical trials, reports are already mixed [159,174,175,176]. Limited studies showed some therapeutic potential for neck pain [165] and chronic traumatic brain injury [193]. Others point that, so far, there are insufficient data to draw firm conclusions on the clinical effect of low power and low optical intensity lasers [176]. One should not forget about laser penetration depths. Scattering and absorption limit light propagation into tissues. In skin, the effective penetration depth at which the incident optical energy drops to ~37% is about 50–100 μm for UV and blue light (λ = 400–450 nm) above 2 μm. The penetration depth of green light (500–550 nm) is about ~200–800 μm. The largest penetration depth ~1–3 mm was found for red and NIR light (600–1350 nm) [159]. Laser penetration depth is one of major challenges of successful clinical application of lasers.

Second group of studies on low power but high optical intensity lasers shows exclusively “negative” biological effects that are not intended to be translated into some clinical application [159,168,170,190,191,192]. Those studies deal with phototoxicity of lasers. In this group of studies some consensus was found. Different research groups showed that high irradiation intensities generate ROS through excited-state reactions of endogenous and exogenous chromophores that have a high potential to damage cellular components [159,168,170,190,191,192]. Numerous studies revealed that high optical intensity low-power laser irradiation induced cell apoptosis or necrosis via the mitochondrial signaling pathway [159,168,170,190,191,192]. The detailed mechanism of phototoxicity induced by high optical intensity low power lasers is still not well understood. However, it seems that researches have no doubts on toxicity exerted by high optical intensity low power lasers.

Described here inconsistencies in the literature are not surprising. Although the biological effects of low power laser radiation have been studied for decades, underlying biochemical mechanisms of laser triggered biological responses remain poorly understood. Moreover, laser photobiomodulation-based therapy might demonstrate a biphasic dose response curve, where low doses appear to have beneficial therapeutic effects while higher doses are harmful (phototoxic) [194,195]. However, there is an unmet need for further methodologically rigorous studies to evaluate the effects of lasers and decipher molecular mechanisms. Such studies should be compared to other laser treatments modalities, different lengths of treatment, wavelengths and dosages.

## 5. Conclusions

In this review we proposed brief, yet comprehensive, discussion on biomedical applications of particular external physical cues, namely high-gradient magnetic fields in nanoparticle-mediated cell responses, non-thermal plasma and laser photobiomodulation. Although all three directions hold great research and clinical promises, they all suffer from relatively same methodological problems. First of all, three research directions retain in the basis a physical cue that contains many variable parameters. Studies of high-gradient magnetic fields in nanoparticle-mediated cell responses have variability in field strength, gradient, frequency and nanoparticle composition. Non-thermal plasma varies on carrier gas, electrode voltage, exposure duration, type of plasma generator (DBD or jet). Laser photobiomodulation is also flexible in a large number of parameters such as the wavelength, fluence, power density, pulse structure, and timing of the applied light. Variable to a large extent physicochemical parameters create a problem of treatment standardization of discussed here physical cues. Therefore, it is very difficult to compare results from different studies. In discussed lines of research, there is a lack of awareness about the guidelines for standardized and transparent reporting of biomedical research. Unification of treatment protocols is an important step that can improve research through increased data quality, better data integration and reusability. Additionally, cell line authentication is still poorly reported. Importantly, research carried out with misidentified cell lines add misinformation to the literature and is likely not to be reproducible [196]. The collection of high-quality data from a large range of cell lines and different labs is essential for establishing scientifically sound grounds for biomedical applications of discussed here external physical cues.

Another challenge is that numerous studies ascribed a sometimes bewildering variety of biological effects in response to high-gradient magnetic fields, non-thermal plasma as well as laser photobiomodulation. We have to identify one simple test biological system and make an explicit prediction. Such approach will help in dissecting the mechanism(s) of action of external physical cues. study. The lack of a clear understanding of the molecular basis of treatment action may contribute to severe side effects [197,198,199]. Thus, we have to continue to study in great details the molecular mechanisms of external physical cues. This will increase the chances for approval of physics-based treatments, save money and time. Additionally, there is a need for clarifying the exact nature of the physical or biochemical stimuli induced by discussed here external physical cues. It is very important that all biologically relevant experimental conditions are reasonably reproducible and consistently repeated. Only then biological effects triggered by high-gradient magnetic fields, non-thermal plasma as well as laser photobiomodulation may mature into established scientific facts.

It is worth noting here, that very often major physics-based medical innovations were completely unpredictable spin-offs from basic science research [1]. Indeed, their translation into clinical practice was not always been as rapid or as straightforward as expected [1]. In this context, there is a need to change research strategy and provide more structured integration between research in the physical and life sciences. Only organization of broad range of scientific expertise in multidisciplinary groups of life and medical scientists and practitioners, physical scientists and bioengineers will boost up research in all three here-discussed directions.

## Figures and Tables

**Table 1:** Characteristics of selected magnetic nanoparticles and magnetic devices suitable for magnetofection.

MNP Core	Surface	M_s_^1^ of the Core (emu/g Iron)	Hydrodynamic Diameter (nm)	ζ-Potential (mV)	Magnetic Device	Ref.
magnetite	PEI ^2^	62	63 ± 36	+55.4 ± 1.6	96-Magnets magnetic plate (OZ Biosciences, cat. no. MF10096)	[15,30]
magnetite	PEI + auroyl sarcosinate	N.E. ^3^	106 ± 38	+27.7 ± 1.8	96-Magnets magnetic plate (OZ Biosciences, cat. no. MF10096)	[15]
iron oxide (γ-Fe_2_O_3_)	N.E.	N.E.	96 ± 1	+57.2 ± 1.7	MidiMACS Separator magnet	[31]
CoFe_2_O_4_	N.E.	89.4	12.5 ± 2.0	N.E.	No data	[32]
iron oxide (γ-Fe_2_O_3_)	PEI	N.E.	121 ± 27	N.E.	Magnetic sheet and neodymium magnet (Magna Co. Ltd.)	[33]
magnetite	PEI, streptavidin	N.E.	N.E.	N.E.	NdFeB magnet 5 mm in diameter	[34]
Mg/Fe layered double hydroxide	citrate	N.E.	~80	−23.2 ± 1.6	No data	[35]
magnetite	BPEI ^4^	~20	50–100	~+2.7	NdFeB magnetic plates (Chemicell, Berlin, Germany)	[36]
magnetite	PLL ^5^	N.E.	N.E.	N.E.	Capacitor discharge impulse magnetizer (Model SUH-1220, Taiwan Ferrite Co., Taiwan)	[37]
magnetite	PEI	N.E.	~50	N.E.	Magnetic field generator ‘Dynamic Marker’ (Stetter-Elektronik, Seeheim-Jugenheim, Germany)	[14]
magnetite	carboxymethyl-dextran	N.E.	~200	N.E.	Magnetic field generator ‘Dynamic Marker’ (Stetter-Elektronik, Seeheim-Jugenheim, Germany)	[14]

^1^ M_s_—saturation magnetization; ^2^ PEI—polyethyleneimine; ^3^ N.E.—not estimated; ^4^ BPEI—branched polyethylenimine; ^5^ PLL—Poly-L-Lysine.

**Table 2:** Overview of recent biomedical applications of magnetic manipulation studies.

Application	Force (pN)	Magnetic Flux Density (mT)	Field Gradient (T/m)	NP Diameter (nm)	Ref.
Notch and E-cadherin receptor activity manipulation	1-47	N.E. ^1^	N.E.	10-30	[67]
Stretching of chromatin for gene transcription upregulation	N.E.	~250	N.E.	4000	[68]
Activation of the Wnt/β-Catenin signaling	N.E.	25–120	N.E.	~300	[69]
Stimulation of filopodia formation and oriented cell division	100,000	25–100	2500–70,000	~150–500	[70]
Mechanical control of inner ear hair cells	0.1	N.E.	1000	20–120	[71]
Control deformations in wild-type *Drosophila* embryonic tissues	60	~200	120	~7.5	[72]
Modulation of cell endocytosis	1–100	1210	~10,000	60	[63]
Enhancement of nanoparticles internalization	~10	~7000	~6000	200	[66]
Control of Rac-GTPase signaling	~10–30	~200	1000–10,000	~500	[73]
Induction of apoptosis	20–40	9	N.E.	~1000	[74]

^1^ N.E.–not estimated.

**Table 3:** Overview of microbial inactivation by non-thermal plasma.

Device	Gas	Voltage	Microorganisms	Main Result	Ref.
Dielectric barrier discharge	He	RF ^1^ voltage at about 45 MHz	*P. aeruginosa* *S. aureus* Bacterial biofilms	ROS/RNS accumulation Membrane depolarization	[108]
Floating-electrode dielectric-barrier discharge (FE-DBD)	Air	low-frequency alternating current (120 V)	*E coli* *S. aureus* *methicillin-resistant Staphylococcus aureus (MRSA)*	Decreasing in viability in both bacterial form (planktonic and biofilm)	[109]
miniFlatPlaSter	Air	7 kV	*S. aureus* *MRSA* *E. coli*	Decolonization of *S. aureus MRSA*, *E. coli* in time-dependent manner	[110]
FlatPlaSter	Air	9 kV	*S. aureus* *MRSA* *E. coli*	Decolonization of *S. aureus MRSA* and *E. coli* in time-dependent manner	[110]
Surface Micro-Discharge (SMD) plasma	Air	10 kV	*E. coli K12* *E. coli* *B. cepacia* *P. aeruginosa* *Gram-positive bacteria* *S. aureus* *MRSA* *S. epidermidis* *E. faecalis* *E. mundtii* *B. cereus* *B. pumilus* *C. difficile* *S. pyogenes* *C. jeikeium* *C. albicans*	The antimicrobial effects of SMD on various vegetative MO and endospores in time-dependent manner	[111]
Plasma jet	Air or Ar	5.5 kV for air 4–5 kV for Ar	*E.coli*	Air plasma: completely rupture of cell walls, inactivation of bacteria by the influence of oxidative stress on peptidoglycans and lipids in the cell wall and membrane	[112]
Terraplasma GmbH, Garching, Germany	Air	6.4 kV and 10 kV	*S. enterica* *S. Typhimurium* *L. monocytogenes*	Analysis of 4 plasma modes several properties of ham colonized by bacteria post treated with plasma as well as after storage	[113]
Discharge plasma device	He	10 kV	*S. aureus* *B. subtilis* *E.coli* *P. aeruginosa*	Dose-dependent decrease in viability of all the bacterial strains, morphological changes after 60 sec of NTP treatment	[114]
Plasma discharge manufactured by Sominex	O_2_, N_2_ and Ar	-	*P. aeruginosa* *S. aureus* *B. subtilis*	Dose- dependent reduction of viability in all three studied vegetative form of bacteria, together with morphologic and structural changes of spores	[115]
Discharge plasma device	He	0.5 kV10 kV	*S. aureus* *B. subtilis* *E.coli* *P. aeruginosa*	Dose-dependent effects of NTP In low-voltage mode activation of programmed cell death after 15 sec In higher-voltage mode more profound damage of bacteria and physical destruction	[116]
DBD system	Air	80 kV	*E. coli* *S.aureus*	Time-dependent inactivation of both bacterial strains increased level of ROS accumulation, Possible mechanism of bacterial inactivation different between gram positive and gram negative bacteria, leakage of membrane in *E. coli* after plasma treatment	[117]
DBD system	Air	18.6 kV	*S. aureus* *P. aeruginosa* *C. albicans*	Rapid inactivation of antibiotic- resistant MO, surface alternation and permeabilization of membrane, depletion of intracellular ATP production, minor changes on DNA and proteins level	[118]
APPJ	N_2_ gas in combination with different flow rates of H_2_O/HNO_3_ solution	2.2 kV	*E. coli*	Effects of plasma treated water (PTW) on *E. coli* viability, effect of chemical composition of PTW importance of NO_2_- and H_2_O_2_ for antimicrobial activity, analysis of oxidative related gene expression, DNA damage and cell morphology upon PTW treatment	[119]
DBD system	Air or argon (Ar)	0.75 kV	*F. oxysporum* *f.sp. lycopersici* spores	Time-dependent cell death (both apoptosis and necrosis) in Fusarium oxysporum f.sp. lycopersici spores post plasma treatment. Reduction of germination ratesWithout any effect of host plant health or growth	[120]
DBD system	Air	80 kV	*P. aeruginosa* quorum sensing (QS)-regulated virulence factors, *E. coli*, *L. monocytogenes* and *S. aureus* for biofilm formation	Significant time-dependent reduction of bacterial biofilm in all three studied strains after both direct and indirect NTP treatment Disintegrated cell walls Decreasing in concentration of (QS)-regulated virulence factors (pyocyanin and elastase)	[121]
DBD system	Air in combination with 90% N_2_ + 10% O_2_	56 kV and 70 kV	*L. monocytogenes* *E.coli*	Time- dependent MO inactivation, increased with higher voltage level Dose-dependent accumulation of DNA damage and impaired membrane integrity Higher resistance of gram-negative *E. coli* in comparison with *L. monocytogenes*	[122]

^1^ RF—radio frequency.
